# A Distinct Class of Antibodies May Be an Indicator of Gray Matter Autoimmunity in Early and Established Relapsing Remitting Multiple Sclerosis Patients

**DOI:** 10.1177/1759091415609613

**Published:** 2015-10-19

**Authors:** Ann J. Ligocki, Jacqueline R. Rivas, William H. Rounds, Alyssa A. Guzman, Min Li, Melania Spadaro, Lauren Lahey, Ding Chen, Paul M. Henson, Donna Graves, Benjamin M. Greenberg, Elliot M. Frohman, E. Sally Ward, William Robinson, Edgar Meinl, Charles L. White, Ann M. Stowe, Nancy L. Monson

**Affiliations:** 1 Department of Neurology and Neurotherapeutics, University of Texas Southwestern Medical Center, Dallas, TX, USA; 2 Institute of Clinical Neuroimmunology, Ludwig-Maximilian-University, Munich, Germany; 3 Department of Immunology and Rheumatology, Stanford University, CA, USA; 4 Department of Immunology, University of Texas Southwestern Medical Center, Dallas, TX, USA; 5 Department of Pathology, University of Texas Southwestern Medical Center, Dallas, TX, USA

**Keywords:** multiple sclerosis, clinically isolated syndrome, autoantibody, B cell, gray matter, myelin tracts

## Abstract

*These authors contributed equally to the work in this manuscript.We have previously identified a distinct class of antibodies expressed by B cells in the cerebrospinal fluid (CSF) of early and established relapsing remitting multiple sclerosis (RRMS) patients that is not observed in healthy donors. These antibodies contain a unique pattern of mutations in six codons along V_H_4 antibody genes that we termed the antibody gene signature (AGS). In fact, patients who have such B cells in their CSF are identified as either having RRMS or developing RRMS in the future. As mutations in antibody genes increase antibody affinity for particular antigens, the goal for this study was to investigate whether AGS^+^ antibodies bind to brain tissue antigens. Single B cells were isolated from the CSF of 10 patients with early or established RRMS. We chose 32 of these B cells that expressed antibodies enriched for the AGS for further study. We generated monoclonal full-length recombinant human antibodies (rhAbs) and used both immunological assays and immunohistochemistry to investigate the capacity of these AGS^+^ rhAbs to bind brain tissue antigens. AGS^+^ rhAbs did not recognize myelin tracts in the corpus callosum. Instead, AGS^+^ rhAbs recognized neuronal nuclei and/or astrocytes, which are prevalent in the cortical gray matter. This pattern was unique to the AGS^+^ antibodies from early and established RRMS patients, as AGS^+^ antibodies from an early neuromyelitis optica patient did not display the same reactivity. Prevalence of CSF-derived B cells expressing AGS^+^ antibodies that bind to these cell types may be an indicator of gray matter-directed autoimmunity in early and established RRMS patients.

These authors contributed equally to the work in this manuscript.

## Introduction

B cells and antibodies are present in both the cerebrospinal fluid (CSF) and the central nervous system (CNS) of patients with relapsing remitting multiple sclerosis (RRMS), as well as clinically isolated syndrome (CIS) patients who are at high risk of developing RRMS (Krumbholz and Meinl, 2014). B cells are involved in RRMS in multiple ways, including antigen presentation and activation of T cells toward antigens in the brain (Harp et al., 2010), production of proinflammatory cytokines (Ireland et al., 2012), and possibly the production of autoantibodies. In addition, depletion of B cells is an effective treatment for many RRMS patients (Hauser et al., 2008).

The most common multiple sclerosis (MS) lesion is characterized by deposition of both antibodies and complement, and patients with this pattern of pathology can improve with plasmapheresis treatment, which removes circulating antibodies (Lucchinetti et al., 2000). In fact, elevated B cells in the CSF correlate with lesion activity on MRI (Cepok et al., 2005), and both increased intrathecal immunoglobulin synthesis (Sellebjerg et al., 2000) and complement activation (Sellebjerg et al., 1998) are also associated with a more aggressive disease course. Sera from MS patients contain antibodies that mediate damage in myelinating cultures containing astrocytes, neurons, and oligodendrocytes (Elliott et al., 2012). Collectively, these findings implicate a role for antibodies in the pathoetiology of MS.

We recently discovered that B cells from the CSF of early and established RRMS patients express a distinct class of V_H_4 antibody genes (Cameron et al., 2009; Rounds et al., 2014). In fact, the prevalence of this distinct class of V_H_4 antibody genes in the CSF-derived B cell pool can support identification of patients who either have or will develop RRMS with 85% to 94% accuracy depending on the sequencing platform used (Cameron et al., 2009; Rounds et al., 2014; Rounds et al., 2015). What identifies this unique subclass of V_H_4 antibody genes is that they have acquired somatic hypermutations (SHMs) at six codons (31b, 40, 56, 57, 81, and 89, Kabat numbering) within the variable region at an excessive rate compared with controls. We call this unusual antibody gene feature the antibody gene signature (AGS) for RRMS.

The purpose of SHM is to create B cells with higher affinity for their antigen. After accumulating SHMs in their immunoglobulin genes, B cells expressing antibodies with higher affinity for their antigen outcompete their sister cells for survival and activation signals. Considering this, the enrichment of the AGS in CSF B cells of RRMS patients could indicate a selection for B cells with affinity for antigens prevalent in the brain. Therefore, we hypothesized that AGS^+^ B cells may express antibodies that bind to targets within the CNS. CNS tissue harbors AGS^+^ B cells (Ligocki et al., 2010), and CSF B cells from RRMS patients represent those that are found in the tissue itself (Owens et al., 2003). Given this, we generated 32 full-length recombinant human antibodies (rhAbs) from single AGS^+^ CSF B cells of 10 early or established RRMS patients and two AGS^+^ CSF B cells from 1 patient with similar symptoms who converted to neuromyelitis optica (NMO).

Immunohistochemistry on both mouse and human brain tissue demonstrated that 30 of the 32 AGS^+^ rhAbs targeted cellular components of the cortex. Immunofluorescence (IFC) confirmed the rhAbs bind either astrocyte cell bodies and processes or neuronal nuclei and, in some cases, bind both cellular targets. This pattern was unique to the AGS^+^ antibodies from early and established RRMS patients, as AGS^+^ antibodies from an early NMO patient did not display the same reactivity. This is the first known description of a distinct class of RRMS-derived antibodies sharing a mutational pattern that targets astrocytes and neurons. The prevalence of B cells expressing AGS^+^ antibodies may be an indicator of gray matter-directed autoimmunity in early and established RRMS patients.

## Materials and Methods

### Patient Sample Acquisition and Processing

CSF was obtained by lumbar puncture from patients recruited in accordance with the University of Texas Southwestern Medical Center (UTSWMC) institutional review board. Patient summary is provided in Table 1. This study includes patient samples as previously published by our group (Cameron et al., 2009; Ligocki et al., 2013) containing established CDMS patients (clinically definite MS) with the relapsing remitting form and early MS patients (CIS) with symptoms of either optic neuritis (ON_CIS_) or transverse myelitis (TM_CIS_). The ON_CIS_ and TM_CIS_ patients all had gadolinium-enhancing lesions at the time of sampling and converted to RRMS subsequent to the time of sampling, with the exception of the one patient who converted to NMO. The samples were stained with fluorescently labeled antibodies and sorted for single CD19^+^ B cells as previously described, into 96-well plates using either the BD FACSAria flow cytometer (Becton Dickinson, San Jose, CA) or the MoFlo High-Performance Cell Sorter (Cytomation, Fort Collins, CO).

**Table 1.** Patient Summary.

Patient number^a^	Diagnosis	Age	Gender	MRI activity	AGS score^b^	% AGS^+^ B cells^c^
1	RRMS	19	F	GD^+^	17.6	22
2	ON_CIS_	40	F	GD^+^	6.1	50
3	ON_CIS_	40	F	GD^+^	10.7	27
4	TM_CIS_	28	F	GD^+^	17.9	18
5	TM_CIS_	20	F	GD^+^	16.7	28
6	TM_CIS_	33	M	GD^+^	22.3	13
7	RRMS	32	F	GD^+^	14.0	14
8	RRMS	44	F	GD^+^	14.0	11
9	RRMS	35	F	GD^+^	15.0	23
10	ON_CIS_	34	F	GD^+^	13.1	25
11	TM_NMO_	57	F	GD^+^	13.8	12

*Note.* AGS = antibody gene signature; GD^+ ^= gadolinium enhancing lesion positive; F = female; M = male; MRI = magnetic resonance imaging; ON_CIS_ = clinically isolated syndrome with optic neuritis symptoms; RRMS = relapsing remitting multiple sclerosis; TM_CIS_ = transverse myelitis; T_NMO_ = transverse myelitis that converted to neuromyelitis optica.

a Overall antibody repertoire data were originally presented for Patient 7 in (Monson et al., 2005), Patients 8 and 9 in (Harp et al., 2007), Patients 4, 5, and 6 in the study by Ligocki et al. (2013) and Patients 1, 3, and 10 in the study by Rounds et al. (2014). ^b^AGS scores were originally presented for Patients 7 to 9 in the study by Cameron et al. (2009) and Patients 1, 3 to 6, and 10 in the study by Rounds et al. (2014). ^c^AGS+ B cells indicate percent of B cells in the CSF of the patient that had 2 or more mutated AGS codons.

### Single-Cell Polymerase Chain Reaction and Genetic Analysis of Variable Heavy and Variable Kappa Genes

After the single cell sort and cell lysis, antibody variable regions were reverse transcribed from messenger RNA, and genetic analyses were performed as previously described (Ligocki et al., 2013). Antibody variable heavy (V_H_) and variable kappa (V_K_) sequences were analyzed and compiled using a Perl program developed at UTSWMC (Ligocki et al., 2010; Ligocki et al., 2013) using IMGT/V-QUEST as the initial source for sequence alignment. AGS scores are determined by calculating a *Z* score as a sum of (replacement mutation frequency % − 1.6%) for each AGS codon (31b, 40, 56, 57, 81, and 89) divided by 0.9%, where replacement mutation frequency percentage is calculated as replacement mutations at a specific codon divided by total replacement mutations (Cameron et al., 2009).

### Cloning and Production of Full-Length Recombinant Human Immunoglobin G Antibodies

Only those CSF B cells expressing the distinct V_H_4 subclass of antibody genes with replacement mutations at two or more of the six AGS codons (31b, 40, 56, 57, 81, and 89) were considered for cloning into full-length expression vectors (i.e., “AGS^+^”; Supplemental Tables 1 and 2). These AGS^+^ rhAbs were cloned from 10 CSF patient repertoires: 9 rhAbs from four established MS patients, 23 rhAbs from six early MS patients, and 2 rhAbs from one early NMO patient, which served as controls for the AGS^+^ rhAbs cloned from the early and established MS patients. Sixty percent of the MS and early MS rhAbs were clonally expanded and were identified by the presence of another V_H_ sequence within the same patient’s antibody repertoire with identical amino acids in the third complementarity determining region (CDR3). The corresponding V_K_ sequence was amplified from the same well as the V_H_ sequence to identify the antibody binding region of the single CSF B cell. Expression vectors for both the Immunoglobin G and Immunoglobin kappa chains and the procedure for production of monoclonal human Immunoglobin G_1_ is well described (Tiller et al., 2008). One additional control rhAb, B1, was cloned from systemic lupus erythematosus (SLE) patient B cells and provided by Dr. Betty Diamond as a construct control that does not bind to mouse brain (Zhang et al., 2009). Production of monoclonal rhAbs and their biotinylation procedure is detailed in the Supplemental Methods.

### Brain Tissue Processing and Immunohistochemistry

Detailed methodology for immunohistochemistry and IFC to detect rhAb binding on brain tissue is provided in the Supplemental Material. Notable differences employed in this current study compared with previously used protocols (von Budingen et al., 2008; Owens et al., 2009) are (a) the usage of 4% paraformaldehyde as a gentle fixative for previously frozen material, rather than paraffin embedding and (b) staining performed on both healthy and diseased white matter (WM) and gray matter (GM) from both mouse and human brain tissues. WM and GM were normal appearing, with the exception of MS plaque tissue. Of note, the presence of lipofuscin, which is typical for mature neurons (Double et al., 2008), is detectable in some of the human brain staining. Also, only the corticospinal subclass of neurons express YFP2.2 in the mice used for IFC (Figure 9; Feng et al., 2000).

### Myelin Oligodendrocyte Glycoprotein, Myelin Basic Protein, and Lysate ELISAs

The rhAbs were tested for binding to myelin oligodendrocyte glycoprotein (MOG), myelin basic protein (MBP), and tissue lysates (brain and kidney) by ELISA using adapted methods (Kinnunen et al., 2013). Detailed methods of rhAb-binding detection are provided in the Supplemental Methods.

### Flow Cytometry of Human Myelin Oligodendrocyte Glycoprotein-Transfected HeLa cells

The rhAbs were tested for binding to hMOG-transfected HeLa cells by fluorescence-activated cell sorter-staining as described (Mayer et al., 2013). Detailed methods are provided in the Supplemental Methods.

### Myelin Array

The rhAbs were tested for binding to a myelin array (Robinson et al., 2003) comprising 406 antigens (375 peptides, 28 proteins, and 3 nucleic acids) plus controls representing major components of the myelin sheath, including MOG and MBP. Detailed methods are provided in the Supplemental Methods.

## Results

### AGS^+^ rhAbs Bind to Cellular Components of the Cortical GM

**Figure 1.**
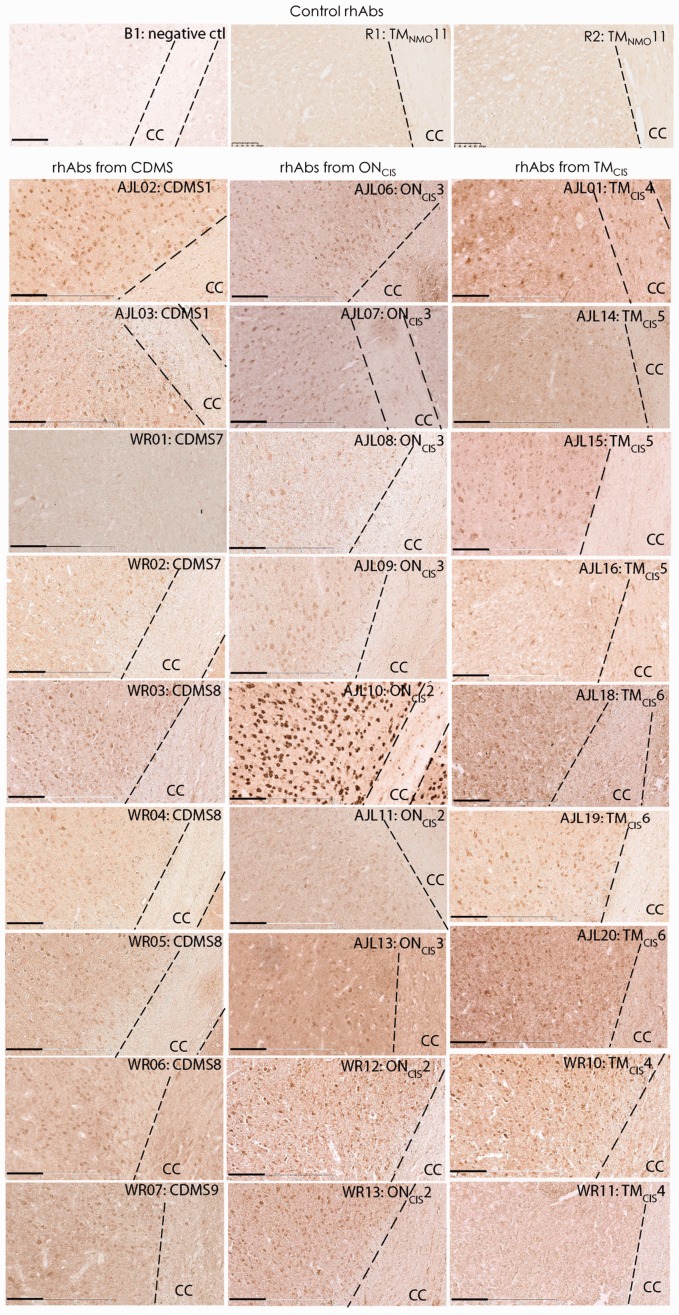
AGS^+^ rhAbs bind to poststroke mouse brain cortex. DAB images are shown at 20× magnification of the cortex and corpus callosum CC (CC within the dotted outline) as indicated. The upper three panels are the negative controls: B1, an rhAb isolated from the brain of a SLE patient with no known reactivity to human or mouse brain tissue; R1, an rhAb isolated from the CSF of an early NMO patient; and R2, an rhAb isolated from the CSF of the same early NMO patient. The nine rhAbs in the first column below these controls are from CDMS patients, the nine rhAbs in the second column below these controls are from ON_CIS_ patients, and the nine rhAbs in the third column below these controls are from TM_CIS_ patients. The rhAb designation, patient type, and patient number are shown in the upper right corner of each image. Data are representative of three coronal sections per rhAb. Scale bar represents 100 µm. AGS = antibody gene signature; CC = corpus callosum; CDMS = clinically definite multiple sclerosis; CSF = cerebrospinal fluid; NMO = neuromyelitis optica; ON_CIS_ = optic neuritis; rhAb = recombinant human antibody; SLE = systemic lupus erythematosus; TM_CIS_ = transverse myelitis.

As MS is considered a demyelinating disease (Hauser and Oksenberg, 2006; Compston and Coles, 2008; Steinman, 2014; Ransohoff et al., 2015), we first sought to determine whether the AGS^+^ rhAbs recognized components of the corpus callosum, which is enriched for myelin tracts. To do this, we utilized brain tissue from a mouse model of transient stroke as a source of inflammation (Stowe et al., 2011), which provided generalized non-antigen-directed inflammation to minimize any potential bias of CNS antigens. 3,3′-Diaminobenzidine (DAB) was used to detect binding to brain tissue of 32 AGS^+^ rhAbs from early and established MS patients and 2 AGS^+^ rhAbs from a patient with NMO. All but 2 of the 32 AGS^+^ rhAbs from early and established MS patients (WR01 and WR11) bound to mouse poststroke brain albeit with a wide range of staining intensity (27 AGS^+^ rhAbs are shown in Figure 1, and the remaining 5 are not shown). However, binding to myelinated tracts in the corpus callosum was either nonexistent or sporadic for all 32 AGS^+^ rhAbs from MS patients. The 2 AGS^+^ rhAbs from an NMO patient (R1 and R2) did not bind to any component of the brain tissue.

**Figure 2.**
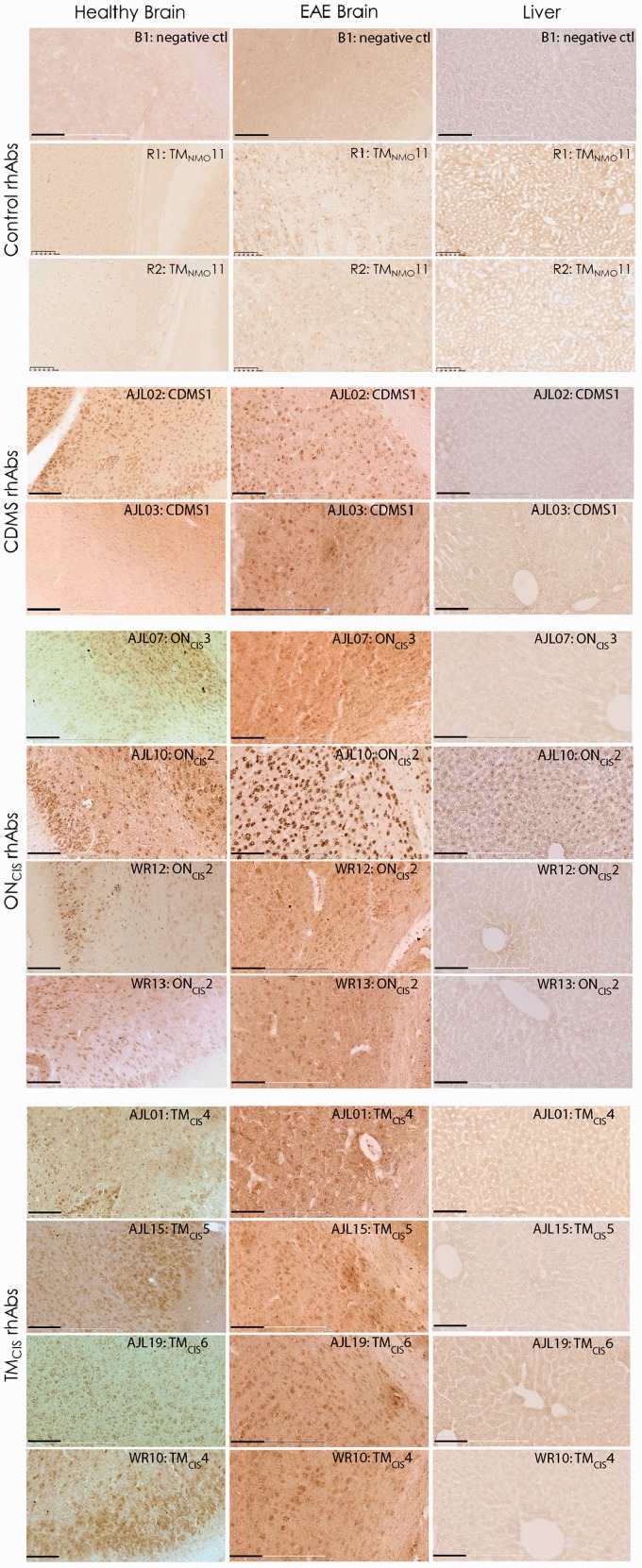
AGS^+^ rhAbs bind to healthy and experimental autoimmune encephalomyelitis mouse brain but not mouse liver. DAB images of negative controls and AGS^+^ rhAbs binding to the cortex and corpus callosum of healthy mouse brain and EAE brain as well as healthy liver tissue are shown at 20× magnification. The upper nine panels are the negative controls (B1, R1, and R2) on these three tissue types. The patient types of the 10 AGS^+^ rhAbs (two from CDMS patients, four from ON_CIS_ patients and four from TM_CIS_ patients) are shown at the left of each panel set and the rhAb designation, patient type, and patient number in the upper right corner of each image. Data are representative of three sections per rhAb. Scale bar represents 100 µm. AGS = antibody gene signature; CDMS = clinically definite multiple sclerosis; ON_CIS_ = optic neuritis; rhAb = recombinant human antibody; TM_CIS_ = transverse myelitis.

Instead, the 30 positively staining AGS^+^ rhAbs that demonstrated reactivity to the mouse stroke brain tissue bound to cellular components of the cortical GM. This binding preference was also confirmed in sections of healthy mouse brain tissue and experimental autoimmune encephalomyelitis (mouse model of MS) brain tissue (Figure 2) using a subset of AGS^+^ rhAbs. Only one of the AGS^+^ rhAbs, AJL10, demonstrated reactivity to healthy mouse liver control tissue (Figure 2). The control rhAbs (B1, R1, and R2) did not bind to either brain or liver tissue (Figures 1 and 2).

### AGS^+^ rhAbs Target Neurons and Astrocytes in the Cortical GM

**Figure 3.**
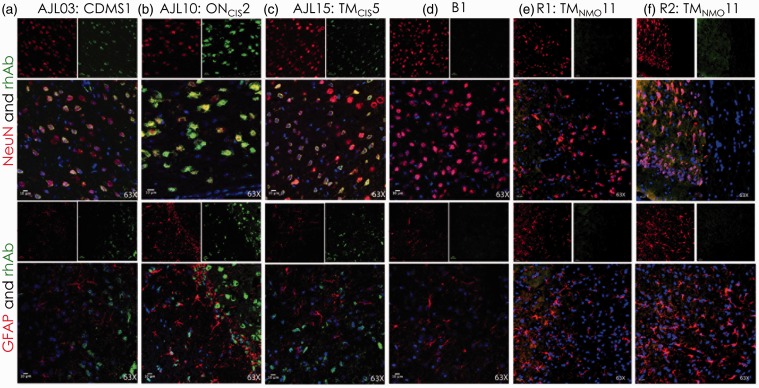
IFC of AGS^+^ rhAbs targeting neuronal nuclei. Confocal images are shown at 63× magnification. The primary rhAb is shown in green in all overlay panels. The experimental rhAbs (AJL03, AJL10, and AJL15) are shown in Panels A to C and the negative control rhAbs (B1, R1, and R2) are shown in Panels D to F. The upper overlay panels include the colocalization marker, NeuN (for neuronal nuclei), shown as red. The lower overlay panels include the colocalization marker, GFAP (for astrocytes), shown as red. The independent red and green channel images are located above each overlay and include the nuclear 4′,6-diamidino-2-phenylindole counterstain. Data are representative of six coronal sections per rhAb. Scale bar represents 10 µm. AGS = antibody gene signature; IFC = immunofluorescence; rhAb = recombinant human antibody.

**Figure 4.**
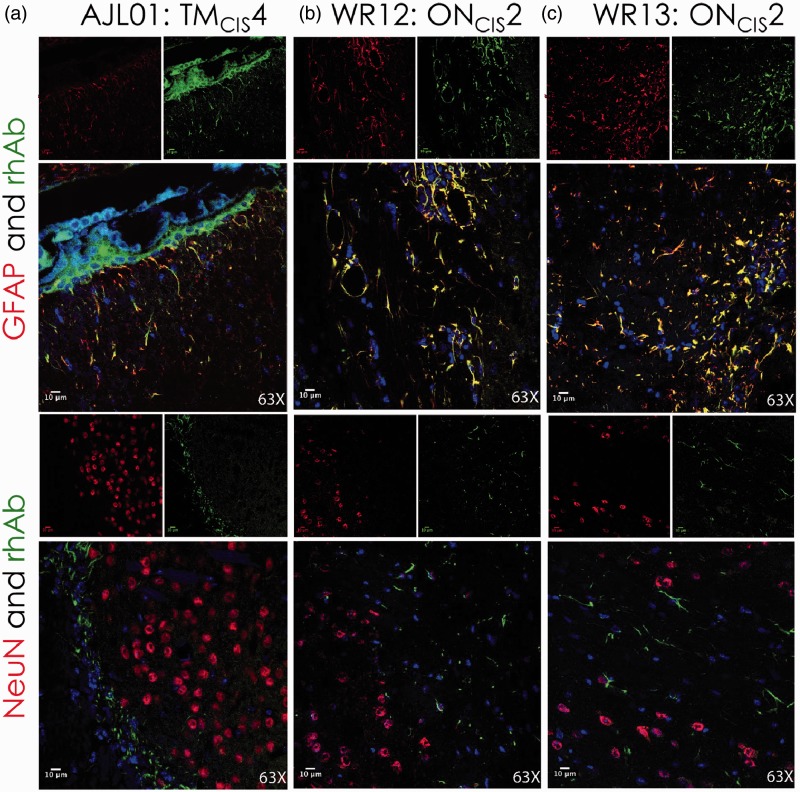
IFC of AGS^+^ rhAbs targeting astrocytes. Confocal images are shown at 63× magnification. The primary rhAb is shown as green in all overlay panels. The experimental rhAbs (AJL01, WR12, and WR13) are shown in Panels A to C. The negative control rhAbs (B1, R1, and R2) are shown in Panels D to F of Figure 3. The upper overlay panels include the colocalization marker, GFAP (for astrocytes), shown as red. The lower overlay panels include the colocalization marker, NeuN (for neuronal nuclei), shown as red. The independent red and green channel images are located above each overlay and include the nuclear 4′,6-diamidino-2-phenylindolecounterstain. Data are representative of six coronal sections per rhAb. Scale bar represents 10 µm. AGS = antibody gene signature; IFC = immunofluorescence; rhAb = recombinant human antibody.

**Figure 5.**
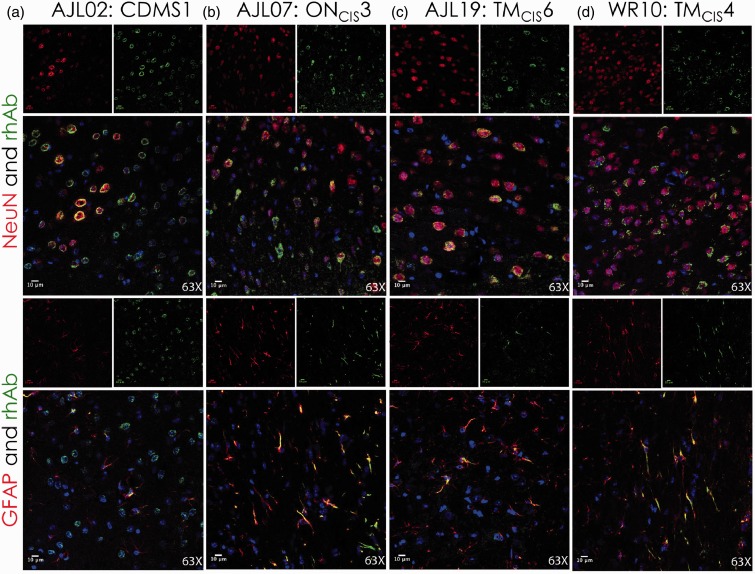
IFC of AGS^+^ rhAbs targeting both neurons and astrocytes. Confocal images are shown at 63× magnification. The primary rhAb is shown as green in all overlay panels. The experimental rhAbs (AJL02, AJL07, AJL19, and WR10) are shown in Panels A to D. The negative control rhAbs (B1, R1, and R2) are shown in Panels D to F of Figure 3. The upper overlay panels include the colocalization marker, NeuN (for neuronal nuclei), shown in red. The lower overlay panels include the colocalization marker, GFAP (for astrocytes) shown in red. The independent red and green channel images are located above each overlay and include the nuclear 4′,6-diamidino-2-phenylindolecounterstain. Data are representative of six coronal sections per rhAb. Scale bar represents 10 µm. AGS = antibody gene signature; IFC = immunofluorescence; rhAb = recombinant human antibody.

Due to the focused cellular binding in the cortical GM by these AGS^+^ rhAbs from MS patients, we hypothesized that the AGS^+^ rhAbs were binding to either neurons or astrocytes in the cortex. Therefore, IFC colocalization experiments were performed on a subset of AGS^+^ rhAbs that demonstrated strong binding by DAB (Figures 1 and 2). NeuN was utilized as a marker for neuronal nuclei, and glial fibrillary acidic protein (GFAP) was utilized as a marker for astrocytes. Figure 3 features three of these rhAbs that bound to neurons but not astrocytes, Figure 4 features three of these rhAbs that bound to astrocytes but not neurons, and Figure 5 features four of these rhAbs that bound both neurons and astrocytes. Negative control IFC using the B1, R1, and R2 rhAbs are shown in Figure 3.

Three of the AGS^+^ rhAbs (AJL03, AJL10, and AJL15) expressed by CSF-derived B cells from three different early MS patients colocalized with neuronal nuclei, as demonstrated in Figure 3 (top panels). Each rhAb contained replacement SHMs at two or more of the six AGS codons (Supplemental Table 1). No additional clones of AJL03 or AJL10 were detected in their respective patients’ CSF, but additional clones of AJL15 were detected. None of these rhAbs cross-reacted with astrocytes as demonstrated by the lack of colocalization with the astrocyte specific antibody, GFAP (Figure 3, bottom panels). The control rhAbs (B1, R1, and R2) did not colocalize with NeuN or GFAP (Figure 3, Panels D–F).

Three different AGS^+^ rhAbs (AJL01, WR12, and WR13) expressed by CSF-derived B cells from early MS patients colocalized with the astrocyte specific antibody GFAP, as shown in Figure 4 (top panels). WR12 and WR13 were from the same patient, but AJL01 was from a different patient. Each rhAb contained replacement SHMs at two or more of the six AGS codons (Supplemental Table 1), and all three rhAbs had additional clones detected in their respective patients’ CSF. None of these rhAbs cross-reacted with neuronal nuclei as demonstrated by the lack of colocalization with NeuN (Figure 4, bottom panels).

Four remaining AGS^+^ rhAbs (AJL02, AJL07, AJL19, and WR10) expressed by CSF-derived B cells from early MS patients colocalized with both neurons (Figure 5, top panels) and astrocytes (Figure 5, bottom panels). Like the previous rhAbs, these rhAbs contained replacement SHMs at two or more of the six AGS codons (Supplemental Table 1), and though they all were cloned from different patients, three of the four rhAbs (AJL07, AJL19, and WR10) had additional clones detected in their respective patients’ CSF.

**Figure 6.**
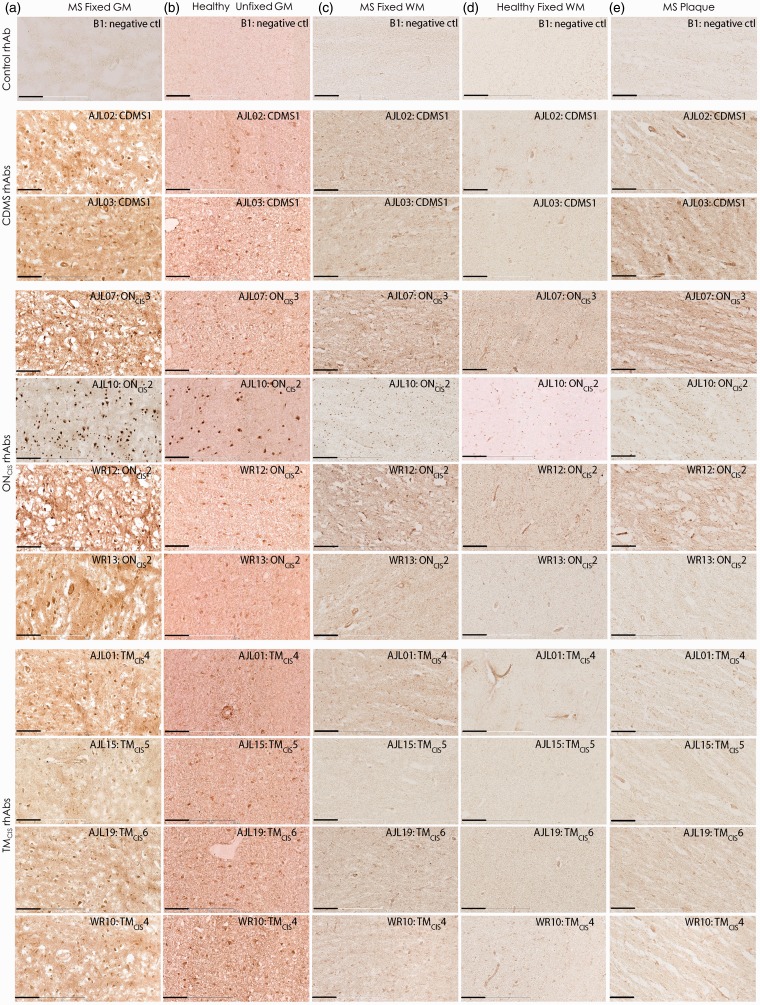
AGS^+^ rhAbs recognize cellular targets in the GM from MS patients and healthy donors but not MS plaques or WM. DAB images are shown at 20× magnification of fixed GM brain tissue from an MS patient (a), GM of unfixed brain tissue from a healthy donor (b), WM of fixed brain tissue from an MS patient (c), WM of fixed brain tissue from a healthy donor (d), and plaque area of fixed brain tissue from an MS patient (e). The rhAb designation, patient type, and patient number are shown in the upper right corner of all figures. DAB data of 10 AGS^+^ rhAbs and the B1 control rhAb are representative of three sections per rhAb. Scale bar represents 10 µm. AGS = antibody gene signature; GM = gray matter; MS = multiple sclerosis; rhAb = recombinant human antibody; WM = white matter.

**Figure 7.**
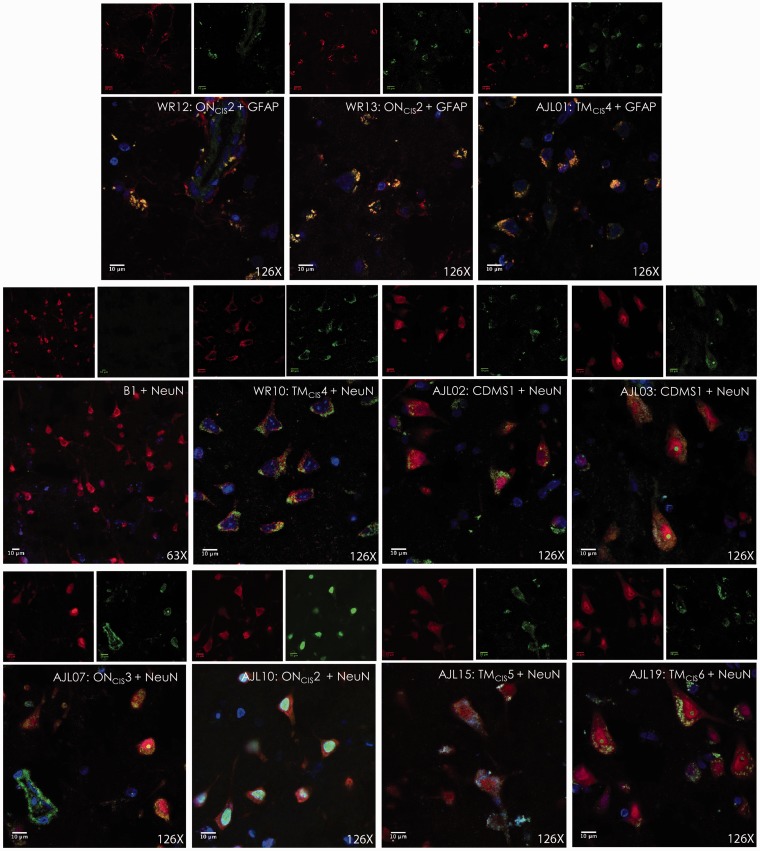
IFC of AGS^+^ rhAbs targeting neurons and astrocytes in GM brain tissue from an MS patient. IFC images are shown at 126× magnification of fixed GM brain tissue from an MS patient. The primary rhAb is shown in green in all overlay panels. The 10 AGS^+^ rhAbs and the negative control rhAb (B1) are shown. The upper row of three overlay panels include the colocalization marker, GFAP (for astrocytes) shown in red, and the lower rows of eight overlay panels include the colocalization marker, NeuN (for neuronal nuclei) shown in red. The independent red and green channel images are located above each overlay and include the nuclear 4′,6-diamidino-2-phenylindole counterstain. The rhAb designation, patient type, patient number and counter-stain antibody (NeuN or GFAP) are shown in the upper right corner of all figure overlays. IFC data are representative of three sections per rhAb. Scale bar represents 100 µm. AGS = antibody gene signature; GM = gray matter; IFC = immunofluorescence; rhAb = recombinant human antibody.

These 10 rhAbs that bound to either neurons or astrocytes in mouse brain tissue were tested for binding of human brain tissue (Figure 6). All 10 AGS^+^ rhAbs recognized cellular targets in the cortical GM from fixed brain tissue of an MS patient (Figure 6(a)) and a healthy donor unfixed cortical GM (Figure 6(b)). However, none of the AGS^+^ rhAbs recognized cellular targets in the fixed WM of an MS patient (Figure 6(c)) or fixed WM of a healthy donor (Figure 6(d)). Minimal recognition of fixed plaque tissue of an MS patient was observed for all AGS^+^ rhAbs (Figure 6(e)). Cellular targets of human GM were identified with colocalization experiments using NeuN and GFAP to replicate what was done in mouse tissue (Figures 3–5). All 10 AGS^+^ rhAbs recognized either neuronal nuclei or astrocytes in human brain tissue as demonstrated by colocalization with either NeuN or GFAP (Figure 7).

### AGS^+^ rhAbs Do Not Bind to Common Myelin Antigens

**Figure 8.**
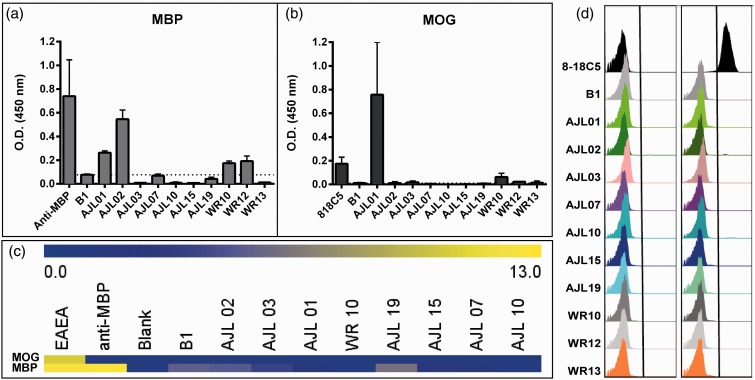
AGS^+^ rhAbs do not bind strongly to common myelin components. (a) and (b) Binding of 10 AGS^+^ rhAbs to common myelin proteins MOG and MBP by ELISA. A dashed line represents the threshold for background signal, as observed with the negative control antibody B1. Commercial anti-MBP (a) and the established anti-MOG antibody 818C5 (b) are included as positive controls. (c) Binding of 8 AGS^+^ rhAbs to myelin-derived peptides demonstrate no reactivity compared to positive controls (an EAE mouse serum antibody pool and anti-MBP). (d) Binding of 10 rhAbs to HEK293 cells mock transfected (left column) or transfected with MOG (right column) demonstrated no reactivity compared to a control MOG antibody (8-18C5). AGS = antibody gene signature; MBP = myelin basic protein; MOG = myelin oligodendrocyte glycoprotein.

We used ELISA, myelin array, and flow cytometry (Figure 8) to evaluate binding of the AGS^+^ rhAbs to the well-known myelin antigens, MBP and MOG (Fraussen, Claes, de Bock, & Somers, 2014). With the exception of AJL01, which was reactive to both MBP and MOG by ELISA, all of the remaining AGS^+^ rhAbs that we tested did not bind MBP or MOG by ELISA (Figure 8(a) and (b)) or by myelin array (Figure 8(c)). Finally, none of the AGS^+^ rhAbs that we tested reacted to MOG expressed on the cell surface of HeLa cells as assessed by flow cytometry (Figure 8(d)).

**Figure 9.**
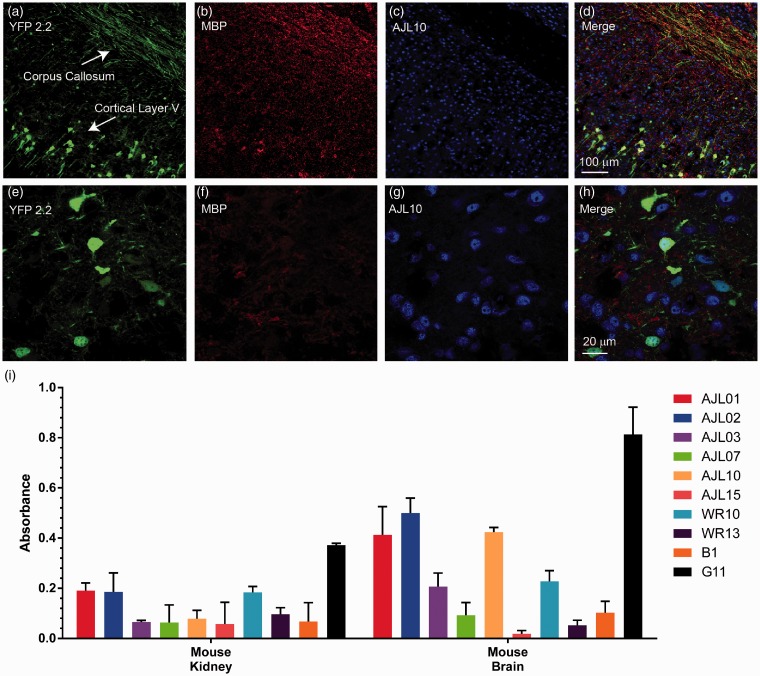
AGS^+^ rhAbs do not bind to myelin tracts, but demonstrate specificity for brain lysate. Panels A to D show a low magnification image (10×) of MBP and AJL10 double IFC staining in brain tissue from YFP2.2 mice, which express soluble YFP in subsets of corticospinal neurons throughout the brain. YFP fluorescence is shown in green (Panel A), MBP in red (Panel B), and AJL10 in blue (Panel C). A merged image containing all three channels is shown in Panel D. Scale bar, 100 µm. Panels E to H show a high magnification image (63×) taken in cortical layer V of MBP and AJL10 double IFC staining in brain tissue from YFP2.2 mice. The immunostaining remained the same as for Panels A to D. A merged image containing all three channels is shown in Panel H. Scale bar represents 20 µm. Data are representative of six coronal sections. The AGS^+^ rhAbs were queried for binding to mouse brain lysate by ELISA (Panel I) using mouse kidney lysate as a control antigen pool. Data are shown as mean and standard deviation of two separate assays with secondary only antibody absorbance subtracted. B1 and G11 are negative and positive controls (respectively) for the brain.

As the majority of AGS^+^ rhAbs did not bind to the common myelin antigens, we next sought to confirm this by staining myelin tracts in the corpus callosum simultaneously with one of the AGS^+^ rhAbs. Brain slices from mice whose corticospinal neurons express YFP2.2 (Feng et al., 2000) were dual stained with a commercial anti-MBP antibody and the AGS^+^ rhAb AJL10 that did not bind to MBP or MOG in the myelin assays (Figure 8). As illustrated in Figure 9, the WM tracts in the corpus callosum were readily identifiable using the anti-MBP antibody colocalized with YFP-expressing axons (Panel B, 10× magnification; Panel F, 63× magnification). AJL10 did not bind to myelin tracts in the corpus callosum, but instead, bound to cellular components in the cortex (Panels C and G), with specific colocalization to layer V neuronal nuclei. Given the prevalence of NeuN and AJL10 colocalization in Figure 3, many of the other nuclei identified may belong to other subclasses of neurons, including interneurons.

To determine whether these AGS^+^ rhAbs bind CNS-specific antigens or antigens expressed in other tissues, ELISAs on tissue lysates from wild-type mice were performed. Many of the AGS^+^ rhAbs colocalized with nuclei, much like antinuclear antibodies (ANAs) found in other autoimmune diseases such as SLE. As deposition of ANAs in the kidneys of SLE patients often leads to glomerulonephritis (acute inflammation of the kidney), we tested our AGS^+^ rhAbs for binding to kidney lysate in comparison to brain lysate. But unlike SLE ANAs, the AGS^+^ rhAbs from these patients showed specificity for brain proteins over kidney proteins (Figure 9(i)). For example, AJL10 bound to mouse brain lysate antigens with five times greater signal compared to mouse kidney lysate.

## Discussion

In this study, we used AGS enrichment as the selection criteria for rhAb generation, as this distinct class of antibodies is unique to B cells in the CSF (Cameron et al., 2009; Rounds et al., 2014; Rounds et al., 2015), and brain tissue (Ligocki et al., 2010) of established and early MS patients with evidence of MRI activity. Previously, our laboratory (Lambracht-Washington et al., 2007) and others (Yu et al., 2006; von Budingen et al., 2008; Owens et al., 2009; Yu et al., 2011) chose antibodies to generate in the laboratory based on whether the CSF-derived B cell had undergone clonal expansion or had differentiated to an antibody secreting plasma cell. Yu et al. identified peptide targets of 10 rhAbs generated from plasma cells of two MS patients, none of which demonstrated homology to any known human proteins, including myelin components (Yu et al., 2006). von Budingen et al. demonstrated that eight of nine rhAbs generated from clonally expanded plasma cells of four MS patients recognized myelin in MS lesion tissue, but reactivity was not to the well-characterized myelin antigens, MBP, MOG, or proteolipid protein (Von Budingen et al., 2008). Interestingly, one of these nine rhAbs recognized astroglia in MS lesion tissue. Finally, Owens and Bennett demonstrated that 53 rhAbs generated from plasma cells of nine MS patients did not recognize individual myelin antigens using multiple immunoassays (Owens et al., 2009). Interestingly, two of these rhAbs recognized neuronal nuclei in MS brain tissue. Taken together, these data indicate that neither myelin-associated or non-myelin antigens can be ruled out as possible targets of antibodies produced by plasma cells in the CSF of MS patients.

For this study, we exclusively chose only those CSF-derived CD19^+^ B cells that expressed AGS^+^ antibodies, which are enriched in the CSF of RRMS patients. Indeed, RRMS patients or patients who will develop RRMS in the future can be identified by the prevalence of AGS^+^ B cells in their CSF (Cameron et al., 2009). We generated 32 AGS^+^ rhAbs from 10 early and established MS patients and 2 AGS^+^ rhAbs from one early NMO patient. DAB staining of the AGS^+^ rhAbs from the early and established MS patients showed binding to cellular components in the cortical GM, with only mild non-myelin cellular staining of satellite astrocytes or oligodendrocytes along the corpus callosum WM. The two AGS^+^ rhAbs from an early NMO patient did not bind to brain tissue. If these rhAbs were strongly targeting WM, there would be an accumulation of staining in the corpus callosum that is composed of highly myelinated axonal tracks, as well as high reactivity in the MBP and MOG ELISA, myelin array, and MOG-focused flow cytometry assays. Instead, we did not observe dominant reactivity by these AGS^+^ rhAbs to any WM component as emphasized by the dual tissue staining of the AGS^+^ rhAb, AJL10, with a commercial anti-MBP antibody (Figure 9).

We did, however, find that the AGS^+^ rhAbs bound to cellular structures in the cortical GM. Interestingly, GM pathology has recently gained appreciation for involvement in MS symptoms and disease progression (Bo et al., 2003; Fisniku et al., 2008; Rudick et al., 2009; Calabrese et al., 2010; Magliozzi et al., 2010; Howell et al., 2011; Schlaeger et al., 2014; Calabrese et al., 2015; Jehna et al., 2015; Kawachi and Nishizawa, 2015; van Munster et al., 2015). More than 50% of all GM-related manuscripts available over the last three decades were published in the last 5 years; decreasing the WM:GM publication ratio to 2.6 compared with 4.9 in the previous two decades. These studies have demonstrated that lesions are more extensive in the GM than in the WM (26.5% vs. 6.5%), such that GM pathology increases with disability and disease length (Bo et al., 2003). Progressive GM loss over time occurs at both the early and established MS stages (Chard et al., 2004; Valsasina et al., 2005), which suggest that the underlying pathology responsible for the loss is not restricted to either later disease stages or the WM myelin tracts. Indeed, others have demonstrated that myelin loss in MS can occur secondary to axonal and neuronal damage (Trapp et al., 1998), and a loss of neuronal precursors can reduce the ability of oligodendrocytes to remyelinate (Einstein et al., 2009). More recently, others have begun to dissect the interaction of T cells with glia in the mouse model of MS (Huseby et al., 2015) and in humans, GM atrophy was higher in patients with evidence of disease activity (Freeman et al., 2015; Nygaard et al., 2015).

As the AGS^+^ rhAbs described in the present study were cloned from B cells in the CSF, which is in close contact with the meninges, the antibodies produced by these AGS^+^ B cells may be strategically located to contribute to GM neuronal damage. For example, antibodies in MS CSF recognize neurofilaments, which comprise the axonal/neuronal cytoskeleton (Fialova et al., 2013). Immunizing mice with neurofilament results in deposition of IgG within neuronal cell bodies and axons and subsequent GM damage (Huizinga et al., 2007). Coincubation of CSF from aggressive MS with neurons *in vitro* induced cell damage, transected axons, and correlated with poor recovery post relapse (Cid et al., 2002).

There were four AGS^+^ rhAbs that did not recognize components of the brain tissue, which was surprising to us, as they all carry two or more replacement mutations in the AGS codons. Two of these AGS^+^ rhAbs that did not bind brain tissue were from early MS patients (WR01 and WR11) and two were from an early NMO patient (R1 and R2). However, we had previously noted (Cameron et al., 2009) that V_H_4^+^ B cells from the CSF of MS patients had a depressed accumulation of SHM at codons 30, 43, 77, and 82. We considered that replacement mutations at these four “cold spots” may extinguish binding to CNS components. Indeed, upon closer examination of these four AGS^+^ rhAbs’ antibody genetics, we noticed that three of them had accumulated replacement mutations at one of more of these cold spots. The AGS^+^ rhAb from an early NMO patient, R1, had not, but it is possible that there are other cold spots in the V_H_4 antibody rearrangements that counteract binding to CNS components that we could not identify due to the small sample size of that data set. Further characterization of the binding specificity of these AGS^+^ rhAbs would need to be done to clarify this issue.

Many of the AGS^+^ rhAbs that were investigated in this study colocalized with neuronal nuclei or appear to be binding intracellular components of neurons and astrocytes. However, unlike ANAs from lupus patients, the majority of these AGS^+^ rhAbs were not reactive to nuclei in liver tissue and had higher affinity for brain extract than kidney extract, indicating their specificity for CNS tissue. There are many known proteins that are expressed specifically in the nuclei of neurons (Maroteaux et al., 1988; Cohen and Roenigk, 1991; Matsuoka et al., 1994; Sakakibara et al., 1996; Coy et al., 2002; Chandrasekar and Dreyer, 2010) and some of these are antigenic targets in other neurological disorders such as paraneoplastic opsoclonus myoclonus ataxia (Buckanovich et al., 1996; Yang et al., 1998) and paraneoplastic encephalomyelitis (Levine et al., 1993; Darnell, 1996; Rauer and Kaiser, 2000) that can cause damage to cells *in vitro* (Graus et al., 1991; Greenlee et al., 1993). Multiple autoimmune diseases have autoantibodies to intracellular antigens and ANAs including systemic sclerosis, SLE, Sjögren’s syndrome, mixed connective tissue disease, and rheumatoid arthritis (Racanelli et al., 2011). While it is currently unknown how autoantibodies to intracellular antigens contribute to disease, there is often a correlation between particular antibodies and involvement of specific organs, disease activity, or even prognosis (Racanelli et al., 2011). Autoantibodies to intracellular antigens may enter cells through receptor-mediated entry (Yanase et al., 1997), endocytosis (Jang et al., 2009), or even electrostatic interactions with membrane proteins (Song et al., 2008) and exhibit cytotoxic effects (Song et al., 2008; Jang et al., 2009).

Despite numerous examples of how antibodies may contribute to MS pathology, it remains unclear whether this distinct class of AGS^+^ antibodies we presented here will display a similar pathological capacity. Nevertheless, these results suggest that this distinct class of AGS^+^ antibodies may be an indicator of cortical GM-directed autoimmunity in early and established MS patients. Understanding early disease progression and identifying novel therapeutic targets will optimize treatment for MS, a disease that globally affects millions of people, while establishing mechanisms for study in other autoimmune diseases of the CNS.

## Summary

B cells in the cerebrospinal fluid of patients with early and established relapsing remitting multiple sclerosis make a distinct class of antibodies that bind to areas of the brain that have not been traditionally investigated in this disease.

## Supplementary Material

Supplementary material

## Supplementary Material

Supplementary material
