# Supplementary material for: A Distinct Class of Antibodies May Be an Indicator of Gray Matter Autoimmunity in Early and Established Relapsing Remitting Multiple Sclerosis Patients
Source: ASN Neuro. 2015 Oct 19;7(5):1759091415609613. doi: 10.1177/1759091415609613 (PMC4710131; doi:10.1177/1759091415609613)
Supplement: Supplementary material [file Supplemental_Tables_613.pdf]

Supplemental Table 1. Patient, Gene, and Staining Overview of the 12 rhAbs used for DAB and IFC

Patient #	Diagnosis	rhAb	V_H; J_H[1]	Vκ; Jκ[2]	Clone	# AGS[3]	V_HJ_H SHM[4]	Mouse Liver[5]	Mouse Stroke/ EAE Brain[6]	Human Fixed/ Unfixed Brain[7]	NeuN[8]	GFAP[9]
1	CDMS	AJL02	4-31; 4	1-39; 2	no	2	8.96%	-	+/+	+/+	+	+
		AJL03	4-39; 1	1-33; 2	no	2	7.96%	-	+/+	+/+	+	-
2	ON_{CIS}	AJL10	4-04; 6	2-28; 5	no	4	12.63%	+	+/+	+/+	+	-
		WR12	4-30; 4	2-28; 4	yes	2	12.44%	-	+/+	+/+	-	+
		WR13	4-30; 4	2-28; 2	yes	2	11.94%	-	+/+	+/+	-	+
3	ON_{CIS}	AJL07	4-59; 4	1-13; 2	yes	3	9.23%	-	+/+	+/+	+	+
4	TM_{CIS}	AJL01	4-34; 3	3-20; 5	yes	3	11.28%	-	+/+	+/+	-	+
		WR10	4-04; 6	3-20; 5	yes	2	4.55%	-	+/+	+/+	+	+
5	TM_{CIS}	AJL15	4-39; 5	2-29; 2	yes	4	7.46%	-	+/+	+/+	+	-
6	TM_{CIS}	AJL19	4-34; 3	3-20; 2	yes	3	8.21%	-	+/+	+/+	+	+
11	TM_{NMO}	R1	4-61; 4	1-39; 2	no	4	10.95%	-	-/-	n/a	-	-
		R2	4-04; 1	3-11; 5	no	2	7.58%	-	-/-	n/a	-	-

Abbreviations: CDMS: clinically definite multiple sclerosis, ON_{CIS}: clinically isolated syndrome- optic neuritis, TM_{CIS}: clinically isolated syndrome-transverse myelitis, NMO: neuromyelitis optica, rhAb: recombinant human antibody, VH: variable heavy chain, JH: variable heavy chain J segment, Vk: variable kappa chain, JK: variable kappa chain J segment, AGS: antibody gene signature, V_HJ_H MF: variable heavy chain rearrangement mutation frequency, EAE: experimental autoimmune encephalomyelitis, NeuN: neuronal nuclei, GFAP: glial fibrillary acid protein

[1] Variable heavy and J segment usage by the rhAb.

[2] Variable kappa and J segment usage by the rhAb.

[3] Number of mutated AGS codons (6 total possible) in the V_H4 gene of the rhAb.

[4] Somatic hypermutation frequency for the heavy chain rearrangement of the rhAb.

[5] Positive (+) or negative (-) staining as determined by DAB staining on mouse liver tissue.

[6] Positive (+) or negative (-) staining as determined by DAB staining on mouse post-stroke brain or EAE tissue.

[7] Positive (+) or negative (-) staining as determined by DAB staining on human brain fixed with 4% paraformaldehyde or unfixed frozen brain. Positive (+) or negative (-) colocalization as determined by the mouse brain tissue IFC experiments.

Supplemental Table 2. Patient, Gene, and Staining Overview of the 22 rhAbs Used Only for Mouse Brain Tissue DAB**.**

Patient #	Diagnosis	rhAb	V_H; J_H[1]	Vκ; Jκ[2]	Clone	# AGS[3]	V_HJ_H SHM[4]	Mouse Stroke Brain[5]
2	ON_{CIS}	AJL11	4-39; 5	3-20; 2	no	3	8.46%	+
3	ON_{CIS}	AJL06	4-30; 4	1-33; 2	no	3	13.93%	+
		AJL08	4-0b; 4	1-5; 2	yes	3	2.53%	+
		AJL09	4-59; 5	1-13; 5	yes	2	7.18%	+
		AJL13	4-59; 4	2-30; 5	yes	3	9.23%	+
4	TM_{CIS}	WR11	4-04; 6	3-11; 4	yes	2	4.55%	-
5	TM_{CIS}	AJL14	4-61; 6	4-1; 2	no	3	10.95%	+
		AJL16	4-39; 5	1-33; 5	yes	3	5.97%	+
6	TM_{CIS}	AJL18	4-31; 5	1-39; 1	yes	5	7.96%	+
		AJL20	4-39; 4	1-27; 4	no	3	9.95%	+
7	CDMS	WR01	4-59; 6	3-20; 2	yes	2	10.26%	-
		WR02	4-30; 6	3-20; 2	yes	2	8.96%	+
8	CDMS	WR03	4-04; 3	3-20; 1	no	3	5.56%	+
		WR04	4-39; 4	3-15; 2	no	3	9.45%	+
		WR05	4-61; 1	1-5; 4	no	4	12.44%	+
		WR06	4-39; 3	1-5; 3	no	3	14.93%	+
9	CDMS	WR07	4-39; 4	3-20; 1	no	4	6.47%	+
10	ON_{CIS}	AJL04	4-59; 5	1-16; 5	yes	3	10.26%	+
		AJL05	4-39; 4	1-16; 5	yes	4	8.46%	+
		AJL12	4-39; 5	3-20; 1	no	3	8.46%	+
		WR08	4-31; 4	2-30; 5	yes	2	7.96%	+
		WR09	4-31; 4	3-20; 2	yes	2	7.96%	+

Abbreviations: CDMS: clinically definite multiple sclerosis, ON_{CIS}: clinically isolated syndrome- optic neuritis, TM_{CIS}: clinically isolated syndrome- transverse myelitis, rhAb: recombinant human antibody, V_H: variable heavy chain, J_H: variable heavy chain J segment, Vk: variable kappa chain, JK: variable kappa chain J segment, AGS: antibody gene signature, V_HJ_H MF: variable heavy chain rearrangement mutation frequency

[1] Variable heavy and J segment usage by the rhAb.

[2] Variable kappa and J segment usage by the rhAb.

[3] Number of mutated AGS codons (6 total possible) in the V_H4 gene of the rhAb.

[4] Somatic hypermutation frequency for the heavy chain rearrangement of the rhAb.

[5] Positive (+) or negative (-) staining as determined by DAB staining on mouse post-stroke brain tissue.
