# Supplementary material for: A Distinct Class of Antibodies May Be an Indicator of Gray Matter Autoimmunity in Early and Established Relapsing Remitting Multiple Sclerosis Patients
Source: ASN Neuro. 2015 Oct 19;7(5):1759091415609613. doi: 10.1177/1759091415609613 (PMC4710131; doi:10.1177/1759091415609613)
Supplement: Supplementary material [file Supplemental_Methods_613.pdf]

Supplemental Methods

Production of monoclonal rhAbs

Human embryonic kidney fibroblast 293T cells were grown to 50-80% confluency in a 10 cm dish in DMEM media supplemented with fetal calf serum (FCS) (Gibco, Life Technologies). The cotransfection of paired cloning vectors corresponding to the IgK and the IgH of a rhAb were mixed (12.5 μg total DNA) with JetPEI solution (Polyplus transfection) and added dropwise to the cells. The plates were incubated in a 5% CO2 water-jacketed incubator (Nuaire, MN, USA) at 37°C in 20mL DMEM media supplemented with ultra-low IgG FCS media (Gibco). Supernatant was harvested and fresh media added on days 3, 5, 7, and 10. Enzyme-linked immunosorbant assays (ELISAs) were used to determine the yield and the concentration of the rhAbs produced in culture. Supernatants were concentrated using the 10 kDa MWCO Amcion Ultra centrifugal filter units (Millipore, MA, USA) following manufacturer's recommendations. A non-transfected cell culture supernatant was confirmed to not contain any IgG above ELISA detection. These concentrated rhAbs were used as primary antibodies for all mouse brain immunohistochemistry.

Biotinylation of monoclonal rhAbs

rhAbs were purified by passing supernatant through a column with a bed of protein G sepharose beads followed by dialysis in PBS and DPBS (Life Technologies). Purity and yield were determined by SDS-page gel stained with coomasie blue and ELISA as described above. Each rhAb was biotinylated using 100 μg of column-purified product and following manufacturer's instructions for the Thermo Scientific EZ-Link Micro NHS-PEG4-Biotinylation kit (Thermo Scientific, MA, USA). These biotinylated rhAbs were used as primary antibodies for all human brain immunohistochemistry.

Processing of frozen brain tissue

Mice were sacrificed 2-3 days post stroke induction as previously described (Stowe et al, Ann Neurol, 2011) and perfused with 4% paraformaldehyde. Brains were extracted and preserved in 4% paraformaldehyde for 48 hrs at 4°C followed by cryoprotection in sequential 15% and 30% sucrose solutions. Liver tissue was extracted as a non-CNS tissue control and processed in the same manner. Brains were also extracted from mice 30 days after induction of EAE using recombinant human MOG as previously described (Lyons et al, Eur J Immunol, 1999). Post-mortem human brain samples were provided by the Human Brain and Spinal Fluid Resource Center (UCLA, Los Angeles, CA). Three samples were used for the studies: white matter (WM) from a healthy control without neurological complications (HC), white matter plaque from a patient with clinically definite multiple sclerosis (MS-P), normal appearing WM from the same multiple sclerosis patient (MS-WM), and normal appearing gray matter (MS-GM). Mean time to sampling from time of death was 16hrs. Upon removing from -80°C, they were preserved similarly to mouse brains with 4% paraformaldehyde for 48 hrs at 4°C followed by cryoprotection in sequential 15% and 30% sucrose solutions. An additional sample of healthy human brain tissue was obtained from the UTSWMC Willed Body Program 24hrs post mortem and prepared for frozen unfixed sectioning. All tissues were embedded in O.C.T. freezing compound by immersing the plastic mold in 2-methyl butane cooled in isopentane for 30 seconds and stored at -20°C (fixed tissue) or -70°C (unfixed tissue) until cryosectioned. Tissue sections (12-16 µm for fixed tissue, 6 µm for unfixed tissue) were cut and mounted on positively charged glass slides using a cryostat (Thermo Scientific MICROM) and frozen at -20°C until used for IHC or IFC. Sections were stained with cresyl violet to validate the integrity and preservation of the tissue.

Diaminobenzidine (DAB)-immunohistochemistry staining of mouse tissue

All animal studies were approved by UT Southwestern Animal Care and Use committee. Tissue sections from Day 2-3 post-stroke, Day 30 post-EAE induction, and healthy mouse brains were subjected to antigen retrieval for 2 minutes using low pH Antigen Unmasking Solution (Vector Laboratories, Burlingame, CA, USA). Endogenous peroxidases were blocked using 3% H_2O_2 solution for 5 minutes at room temperature and then washed. The sections were blocked with 3% normal goat serum in PBS for 10 minutes at room temperature, washed with PBS, and then were incubated overnight at 4°C with 1 µg rhAb (10 ng/µL) per brain slice. The next day, sections were washed and probed with a biotinylated secondary goat anti-human IgG Fc antibody followed by incubation with the avidin-peroxidase solution following manufacturer's instructions (Vector Laboratories, Burlingame, CA, USA). The tissues were incubated with DAB solution according to manufacturer's instructions (Vector Laboratories, Burlingame, CA, USA). The slides were dehydrated and cleared with sequential washes in increasing percentages of EtOH, from 70% to 100%, with two final washes in xylenes. Slides were mounted with a permount:xylene solution and imaged using a 40x brightfield lens on the NanoZoomer (Hammatsu, Japan). Images were visualized using NDP.view software (Hammatsu, Japan) and 20x images were exported for visualization and adjustments to brightness and contrast were done with ImageJ software (NIH, USA).

DAB-immunohistochemistry staining of human tissue

Initial processing of the fixed human brain tissue sections remained the same as the mouse tissue. The unfixed human brain tissue sections were not subjected to the initial step of antigen retrieval. After blocking with 3% normal goat serum in PBS, an additional blocking step was performed with BloxAll for 10 minutes at room temperature (Vector Laboratories, Burlingame, CA, USA). Due to the presence of IgG deposits even in healthy brain and as an artifact of post-mortem tissue preparation, the rhAbs used in the mouse brain IFC were biotinylated to eliminate the need for a species specific secondary antibody.

Fixed and unfixed brain tissue was incubated overnight at 4°C with 1 μg biotinylated-rhAb (10 ng/μL) per brain slice. The next day, these biotinylated-rhAbs were detected without a secondary antibody and instead with ABC reagent alone (Vector Laboratories, Burlingame, CA, USA). Dehydration, clearing, mounting, and visualization of the human tissue followed the same procedure as the mouse tissue.

IFC staining of mouse tissue

Tissue sections were subjected to antigen retrieval for 2 minutes using low pH Antigen Unmasking Solution (Vector Laboratories, Burlingame, CA, USA). The sections were blocked with 1% normal goat serum and 1% Tween-20 in PBS for 1 hour at room temperature. Most of the rhAbs were diluted in blocking solution. Pierce Immunostain Enhancer (Thermo Scientific) was used as the diluent for the primary rhAb incubation as well as the secondary Alexa Fluor488 for the following two rhAbs: AJL03, AJL15. Slides were washed with PBS, and then incubated overnight at 4°C with 1μg rhAb (10 ng/μL) per brain slice. Next day, the sections were washed and incubated for 1 hour at room temperature with the secondary antibody Alexa Fluor 488 goat anti-human IgG Fc (Life Technologies). Then a colocalization marker, either GFAP (Abcam) or NeuN (Chemicon) were used at 1:1000 and 1:100 dilutions respectively, was incubated for 1 hour at room temperature and then incubated for an additional hour with the appropriate secondary antibody Alexa Fluor 594 anti-rabbit IgG Fc for GFAP or Alexa Fluor 594 anti-mouse IgG Fc for NeuN detection (Life Technologies). Next, the stained tissue sections were incubated for three minutes with DAPI (1:1000) as a nuclear counterstain (Life Technologies). The sections were washed and wet mounted with Fluoro-Gel (Electron Microscopy Diatome). Slides were viewed with a fluorescent Leica TCS SP5 confocal microscope (Leica microsystems) and viewed and adjusted in brightness and contrast using ImageJ software (NIH, USA).

IFC staining of human tissue

Initial processing of the human brain tissue sections remained the same as the mouse tissue above. After the initial blocking, endogenous biotin was blocked per manufacturer's instructions using the streptavidin-biotin blocking kit (Vector Laboratories, Burlingame, CA, USA). Pierce Immunostain Enhancer (Thermo Scientific) was used as the diluent for the primary rhAb incubation as well as the secondary Alexa Fluor 488 for all human tissue IFC. Slides were washed with PBS, and then incubated overnight at 4°C with 2μg rhAb (20 ng/μL) per brain slice. Next day, the sections were washed, and incubated for 1.5 hours at room temperature with the secondary antibody Alexa Fluor 488 goat anti-streptavidin (Life Technologies). The colocalization with either GFAP or NeuN, DAPI counterstain, mounting and visualization followed the same procedure as the mouse brain tissue.

MOG and MBP ELISAs

Pre-treated ELISA plates (Fisher) were coated with 10 μg/ml of recombinant human MOG (produced in-house) or human MBP (Sigma-Aldrich) in 1% BSA (Sigma-Aldrich) in PBS overnight at 4˚C. After washing and blocking plates, they were incubated overnight at 4˚C with 1 μg/ml of rhAbs. The next day, plates were washed and probed with 100 ng/ml of biotinylated goat-anti-human IgG Fc (eBioscience) followed by a 1:2000 dilution of streptavidin-HRP (BD Biosciences). Plates were washed and developed with TMB substrate (eBioscience). Absorbance was measured with an Epoch Microplate Spectrophotometer (BioTek) at 450 nm.

Myelin Array

This methodology has been fully described elsewhere (Robinson et al, Nat Biotechnol., 2003). rhAbs were tested for binding to a myelin array comprised of 406 antigens (375 peptides, 28 proteins, 3 nucleic acids) plus controls representing major components of the myelin sheath, including MOG and MBP by the Robinson laboratory. A robotic array printer was used to print the antigens onto SuperEpoxy 2 Protein

Substrates (ArrayIt Corporation) with 4 replicates of each antigen. The arrays were blocked overnight at 4°C with 3% FCS in PBS, then incubated with 300 µL of 3 µg/mL dilutions of the rhAbs for 1 hour at 4°C. Arrays were washed and 1:2500 dilutions of goat-anti-human IgGconjugated to cyanine 3 (Jackson Immunoresearch) were incubated for 45 minutes at 4°C. Arrays were then washed, spun dry and read with a GenePix 4400 A scanner (Molecular Devices). GenePix Pro 7.0 software (Molecular Devices) was used to analyze the images and determine median pixel intensity, while statistical analyses were done with Significance Analysis of Microarrays (SAM) (developed by Dr. Robert Tibshirani, Stanford University).

Flow Cytometry of hMOG Transfected HeLa cells

This methodology has been fully described elsewhere (Mayer et al, J Immunol, 2013). Full-length hMOG was cloned into the pEGFP-N1 plasmid (CLONTECH Laboratories, Mountain View, CA) and transfected into HeLa cells using Lipofectamine® 2000 transfection reagent (Invitrogen). Expression of hMOG was confirmed by staining with the 8-18C5 monoclonal antibody. Antibodies were tested on 100,000 cells at a concentration of 16,28 ng/µl and incubated for 45 minutes at 4˚C, followed by 3 washes with FACS buffer (1% FCS in PBS). Cells were then given a 1:500 dilution of biotin-SP–conjugated goat anti-human IgG (Jackson ImmunoResearch) or biotin-SP-conjugated goat anti-mouse IgG (Jackson ImmunoResearch) for 30 minutes at 4˚C followed by 3 washes and a 1:2000 dilution of streptavidin-Dy light 649 (Jackson ImmunoResearch). Lastly cells were suspended in a 1:500 dilution of propidium iodide to distinguish live and dead cells.

MBP+AJL10 double immunostaining

Brain cryosections (14 µm thickness) were prepared from PFA-fixed YFP2.2 mice (10 months old) (Feng et al., 2000) and stored at -80°C. Slides were allowed to warm to room temperature and rehydrated in PBS. Antigen retrieval was performed for 2 minutes at 95°C in citrate buffer (Vector Labs). The tissue

was permeabilized by incubating in PBS+0.2% (v/v) Triton X-100 for 15 minutes at room temperature. Nonspecific antibody binding was inhibited by incubation in blocking solution (10% normal goat serum, 5% (w/v) BSA, 0.025% Triton X-100 in PBS) for at least 1 hour at room temperature. Primary antibodies were diluted in blocking solution and incubated concurrently overnight at 4°C. Mouse monoclonal antibodies against myelin basic protein (MBP; #SMI-99, Covance) were diluted 1:500 and human AJL10 rhAb was used at 10 ng/µL. The tissue was washed 3 times for 10 minutes each in PBS+0.025% Triton X-100 at room temperature. Fluorophore-conjugated goat anti-mouse and goat anti-human secondary antibodies (GαM AlexaFluor 594 and GαH AlexaFluor 647, Invitrogen Molecular Probes) were diluted 1:500 in blocking buffer and incubated on the sections for 1 hour in the dark at room temperature. The slides were washed 3 times for 10 minutes each in PBS+0.025% Triton X-100 at room temperature, mounted in ProLong Gold anti-fade mounting medium with DAPI (Invitrogen Molecular Probes) and allowed to dry for at least 24 hours in the dark at room temperature. Confocal images were collected at 10X and 63X magnification using a Zeiss LSM510 microscope (UTSW Live Cell Imaging Facility) using 488nm, 543nm, and 633nm laser lines and appropriate filter sets. Images were processed including adjustment of brightness and contrast of the complete images using Zeiss Zen Lite software and cropped in Adobe Photoshop. The figure was prepared in Adobe Illustrator.

Mouse Brain and Kidney Lysate ELISAs

Healthy B6 mice were euthanized and profused with PBS. Brain and kidney tissue was extracted and homogenized in ice cold lysate buffer (150 mM NaCl, 50 mM Tris HCl at pH 8.0 with 1% Triton X, 1% Nonidet P40 and cOmplete protease inhibitor cocktail (Roche). Tissue homogenate was centrifuged for 20 minutes at 16,000 rpm at 4˚C to remove large particulate matter. Supernatant was collected and incubated with a mixture of protein A and G beads (Life Technologies) overnight with gentle rotation, and again with fresh beads for an additional 4 hours to remove antibodies from the lysate. Total protein concentration was determined with a BCA kit (Thermo Scientific) and lysate was aliquoted and frozen at -80˚C. Pre-treated ELISA plates (Fisher) were coated with 5 µg/ml of tissue lysate in biocarbonate buffer

overnight at 4˚C. Plates were washed twice with PBST and blocked with 10% BSA in PBS for 2 hours at room temperature. Plates were washed again and 10 μg/ml of in-house rhAbs (in 5% BSA in PBS) were incubated overnight at 4˚C. Plates were washed 5 times and 100 ng/ml of biotinylated goat-anti-human IgG Fc (eBioscience) was added for 2 hours at room temperature. Plates were washed 5 times and a 1:2000 dilution of streptavidin-HRP (BD Biosciences) was added for 1 hour at room temperature. Plates were washed a final 5 times and developed with TMB substrate (eBioscience) and neutralized after 1 minute with 1M HCl. Absorbance was measured with an Epoch Microplate Spectrophotometer (BioTek) at 450 nm.
